# Enhancement of the Anti-Inflammatory Activity of NSAIDs by Their Conjugation with 3,4,5-Trimethoxybenzyl Alcohol

**DOI:** 10.3390/molecules27072104

**Published:** 2022-03-24

**Authors:** Paraskevi Tziona, Panagiotis Theodosis-Nobelos, Georgios Papagiouvannis, Anthi Petrou, Chryssoula Drouza, Eleni A. Rekka

**Affiliations:** 1 Department of Pharmaceutical Chemistry, School of Pharmacy, Aristotelian University of Thessaloniki, 54124 Thessaloniki, Greece; tzionap@gmail.com (P.T.); anthi.petrou.thessaloniki1@gmail.com (A.P.); 2 Department of Pharmacy, School of Health Sciences, Frederick University, Nicosia 1036, Cyprus; hsc.np@frederick.ac.cy (P.T.-N.); hsc.pag@frederick.ac.cy (G.P.); 3 Department of Agricultural Sciences, Biotechnology and Food Science, Cyprus University of Technology, Lemesos 3036, Cyprus; chryssoula.drouza@cut.ac.cy

**Keywords:** non steroidal anti-inflammatory drugs, anti-inflammatory derivatives, cyclooxygenase inhibition, molecular docking, lipoxygenase inhibition

## Abstract

The synthesis of derivatives of three nonspecific COX-1 and COX-2 inhibitors, ibuprofen, ketoprofen, naproxen is presented. These acids were connected via an amide bond with an amino acid (L-proline, L-tyrosine, and beta-alanine) used as a linker. The amino acid carboxylic group was esterified with 3,4,5 trimethoxybenzyl alcohol. The activity of the novel derivatives was examined in vivo on carrageenan-induced inflammation, and in vitro, as cyclooxygenase and lipoxygenase inhibitors. It was found that the new compounds were more potent anti-inflammatory agents than the parent drugs. Thus, the ibuprofen (**21**) and ketoprofen (**16**) derivatives reduced rat paw edema by 67 and 91% (the reduction by the relevant NSAIDs was 36 and 47%, respectively). They inhibited COX-2 more than the starting drugs (**21** by 67%, ibuprofen 46%, **19** by 94%, ketoprofen 49%). Docking of compounds on the active sites of COX-1 and COX-2 reflects their in vitro activity. Thus, **19** adopts an unfavorable orientation for COX-1 inhibition, but it binds effectively in the binding pocket of COX-2, in agreement with the absence of activity for COX-1 and the high inhibition of COX-2. In conclusion, the performed structural modifications result in the enhancement of the anti-inflammatory activity, compared with the parent NSAIDs.

## 1. Introduction

Inflammation is a defensive reaction of the organism against various invaders. Acute inflammatory reaction is usually followed by repair and healing processes. However, if acute inflammation is not resolved, it may become chronic, causing progressive tissue damage and inducing serious complications [1]. Inflammation is implicated in several pathologic conditions such as asthma, rheumatoid arthritis, atherosclerosis, metabolic syndrome, neurodegenerative diseases, and cancer [2]. The development of boron delivery agents constitutes an emerging technique for the treatment of diseases such as cancer and rheumatoid arthritis [3].

Non-steroidal anti-inflammatory drugs (NSAID), divided into the non-selective, cyclooxygenase (COX) -1 and -2 inhibitors and the selective COX-2 inhibitors, are among the most broadly prescribed drugs for the treatment of inflammatory conditions. However, adverse reactions from the gastrointestinal, renal, and cardiovascular systems limit their long-term application [4]. In addition, reactive oxygen species (ROS) and oxidative stress are implicated in inflammation. Therefore, novel approaches have arisen, such as dual cyclooxygenase/5-lipoxygenase inhibitors, synthetic lipoxins, antioxidants, nitrogen monoxide-, and hydrogen sulfide-releasing NSAIDs [5].

The drug-binding sites in both COX enzymes are almost identical, with the exception of an increased cavity volume, creating a side pocket in COX-2 [6,7]. It has been reported that structural modifications of the non-selective indomethacin lead to bulkier molecules with increased selectivity for COX-2 [8].

The multiple compound approach is a method of rational drug design aiming to multiple targets by one molecule. Linking two or more pharmacophores into a single molecule may lead to compounds with improved pharmacodynamics and/or pharmacokinetics [9].

**Figure 1:**
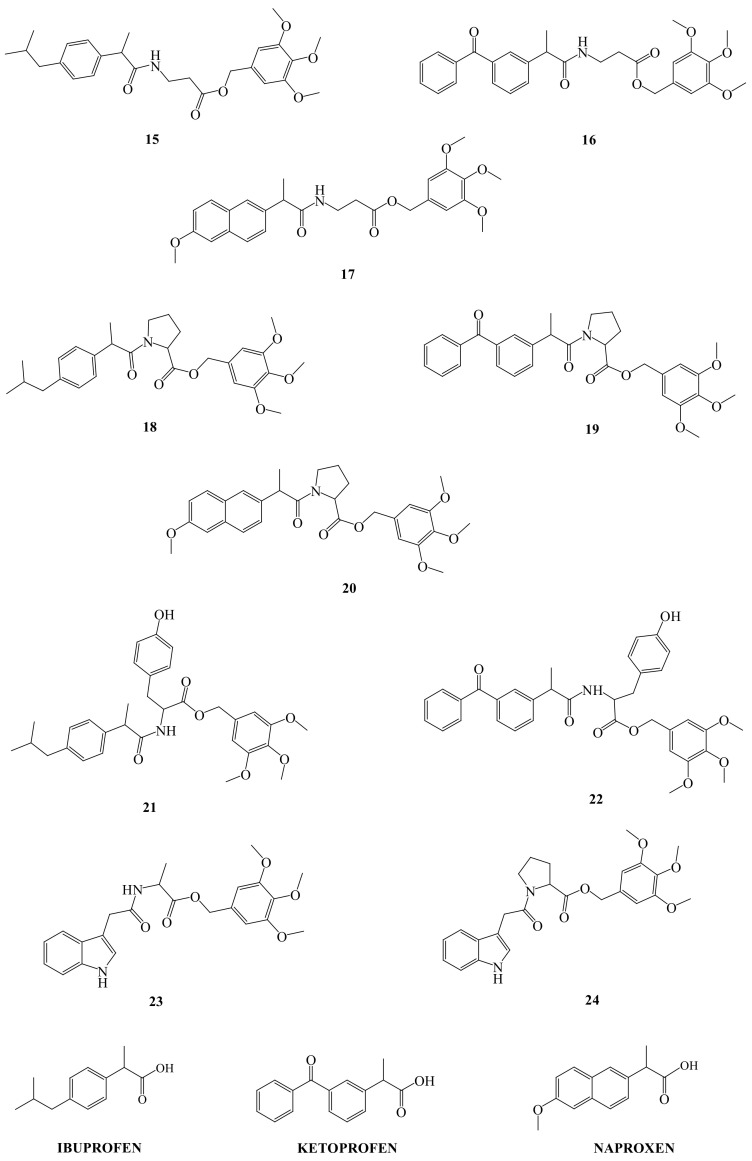
Structures of synthesized derivatives and of ibuprofen, ketoprofen, and naproxen.

In this investigation, the synthesis of derivatives of three well-known nonspecific COX-1 and COX-2 inhibitors, ibuprofen, ketoprofen, naproxen is presented. The above acids are connected via an amide bond with an amino acid moiety (L-proline, L-tyrosine, and beta-alanine) used as a linker. The amino acid carboxylic group is further esterified with 3,4,5 trimethoxybenzyl alcohol (Figure 1).

We have reported that amides of NSAID with amino acids, such as cysteine [10] or L-proline [11] are more potent anti-inflammatory agents than the parent NSAID, with low gastrointestinal toxicity, partly due to their reduced acidic character. Furthermore, we have shown that the anti-inflammatory activity of those compounds was due to the whole molecule and not to the in vivo hydrolysis products, since the administration of an equimolar mixture of the relevant NSAID and amino acid (given as ethyl ester) resulted in lower anti-inflammatory activity than that of the test compounds [10].

Trimethoxybenzyl alcohol was selected because the 3,4,5-trimethoxybenzyl moiety has been encountered in compounds with anti-inflammatory activity, acting as selective COX-2 inhibitors [12], as well as in gastroprotective agents [13,14]. It has also been found that direct esterification of 3,4,5-trimethoxybenzylalcohol with either ibuprofen or ketoprofen enhanced their anti-inflammatory activity [2]. Two more compounds (Figure 1) were synthesized from indole-3-acetic acid, part of indomethacin molecule, via an amide bond with either L-proline, or alpha-alanine, and esterified with 3,4,5-trimethoxybenzyl alcohol.

By the mentioned modifications, the final compounds become quite bulky and are expected to become more lipophilic than the starting NSAID. Thus, an increase of the anti-inflammatory action is expected. These modifications may contribute to an increased inhibition of COX-2, the inducible form of cyclooxygenases, during the inflammatory reaction, and to a lower inhibition of COX-1, thus reducing side effects, such as gastrointestinal and renal toxicity.

The biological activity of the novel derivatives was examined in vivo on carrageenan-induced rat paw edema, as well as in vitro, as cyclooxygenase and lipoxygenase inhibition. The synthesized structures were subjected to in silico docking study for molecular interaction analysis with cyclooxygenases and lipoxygenase. Furthermore, the stability of one derivative in water and plasma was examined, in a preliminary experiment. The derivatives were also tested for their antioxidant capacity.

## 2. Results and Discussion

### 2.1. Synthesis

**Scheme 1:**
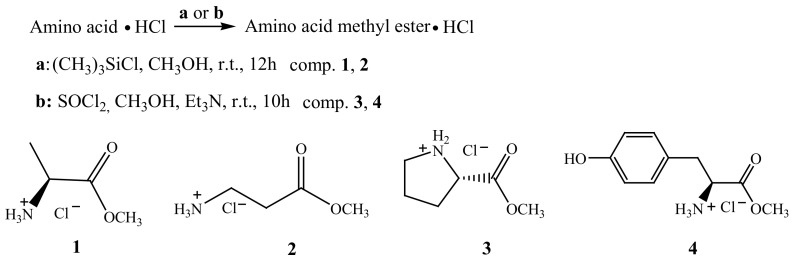
Synthesis of amino acid methylester hydrochlorides.

The starting amino acid methyl esters (as hydrochlorides) were prepared from the amino acids with methanol in the presence of either trimethylchlorosilane [15], or thionyl chloride, [16] in good yields (Scheme 1).

**Scheme 2:**
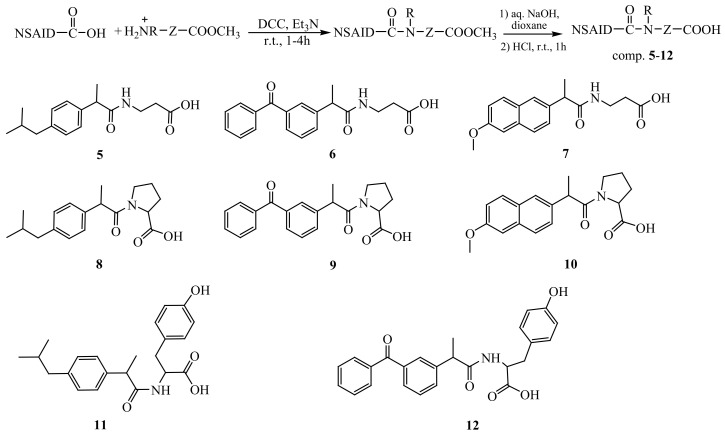
Synthesis of the intermediate amides of NSAID with amino acids.

Intermediate amides of the NSAID were synthesized from the amino acid methyl esters using N,N′-dicyclohexylcarbodiimide (DCC) in the presence of trimethylamine (Et_3_N), followed by hydrolysis with aqueous 5% NaOH and acidification to form the corresponding acid, with minor differences in the reaction time and yields (68–98%) (Scheme 2).

Amides of indole-3-acetic acid with alpha-alanine (compound **13**) and L-proline (compound **14**) were synthesized applying the same method.

**Scheme 3:**
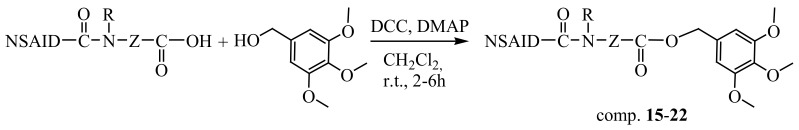
Synthesis of the final compounds.

The final compounds were synthesized by esterification of the above acids with 3,4,5-trimethoxybenzyl alcohol, using DCC in the presence of 4-dimethylaminopyridine (DMAP) (Scheme 3 and Figure 1) [17] with yields 43–80%. The spectroscopic (IR, ^1^H-NMR, ^13^C-NMR) studies and chemical analysis (C, H, N) were carried out to characterize the new compounds and support their molecular structures.

### 2.2. In Vivo Studies

#### Effect on Carrageenan-Induced Rat Paw Edema

Carrageenan-induced inflammation model has significant value for evaluating anti-inflammatory agents [18]. Carrageenan administration causes an acute and local inflammatory response. In the early phase (0–1 h), bradykinin, histamine, tachykinins, complement and reactive oxygen and nitrogen species are involved, whereas prostaglandins and various cytokines such as IL-1β, TNF-α are released in the second phase, more than one hour after administration [19]. The effect of the synthesized compounds on acute inflammation, 3.5 h post injection, applying the carrageenan paw edema model, as well as the anti-inflammatory activity of ibuprofen, ketoprofen, naproxen and indole-3-acetic acid, used as reference compounds, are shown in Table 1.

The results of the Table 1 demonstrate that all compounds were at least of the same activity and up to twice more active than the starting NSAID. It is interesting that, while indole-3-acetic acid is inactive, compound **23** showed a considerable anti-inflammatory activity.

In order to examine the contribution of the trimethoxybenzyl alcohol moiety to activity, the methyl esters of compounds **5**, **8**, **13**, and **14** (**5a**, **8a**, **13a**, and **14a**) were evaluated for anti-inflammatory activity, and the results are given in Table 2.

The methyl esters **5a** and **8a** reported in Table 2 are less active than ibuprofen and the final **15** and **18**. However, the proline derivative **8a** is more active than **5a** and the same order is followed for **18** and **15**. In general, it seems that if a given methyl ester is active, then the corresponding 3,4,5-trimethoxybenzyl ester is even more active, e.g., **13** vs. **23**, compared to **14** vs. **24**.

### 2.3. In Vitro Studies

#### 2.3.1. Inhibition of Cyclooxygenase (COX) Activity (Isoforms **1** and **2**)

Cyclooxygenase catalyzes the conversion of arachidonic acid to prostaglandins, important inflammatory mediators, also significant for gastric mucosa protection, platelet aggregation, and kidney function. COX-1, the constitutive form, participates in the maintenance of cellular homeostasis, whereas COX-2, the inducible isozyme in response to inflammatory stimuli, leads to augmented prostaglandin release.

The effect of the examined compounds as well as that of ibuprofen, ketoprofen, and naproxen, used as reference compounds, (concentration 0.05 μM) on both COX isoforms has been examined. The concentration of the substrate, arachidonic acid, was 0.1 μM. Results are shown in Table 3, together with the molecular volume (MV) of compounds.

The results of Table 3 confirm that ibuprofen, ketoprofen, and naproxen are better inhibitors of COX-1 than of COX-2 [20]. On the contrary, ibuprofen derivatives **18** and **22** and ketoprofen derivatives **16** and **19** inhibited COX-2 more than COX-1. Compound **18** could be considered as a selective inhibitor of COX-2 and compound **19** is a very potent inhibitor of both, COX-1 and COX-2, with a very significant activity on COX-2, 94%. It seems that increase of the molecular volume of the compounds favors their interaction with COX-2; however, there should be an upper limit to this increase, since compound **22**, with the highest molecular volume, lacks any inhibitory activity on COX isoforms. Naproxen derivatives **17** and **20** had almost no effect on any enzyme, however, naproxen itself was found to be a weak inhibitor of COX-2 with a mild activity on COX-1.

From Table 1 and Table 3 it can be realized that inhibition of COX-2 could be correlated with the anti-inflammatory activity of compounds. Thus, compounds **17** and **20**, with no or low COX-2 inhibition, are weak anti-inflammatory agents. On the contrary, compounds inhibiting COX-2 more than 50% (**16**, **18**, **19**, **21**) are the most potent anti-inflammatories, as they reduce edema from 49% to 91%. Interestingly, **22**, with no detectable COX inhibition, is a quite strong anti-inflammatory compound. This could be explained considering that, besides prostaglandins, various inflammatory mediators and cytokines are involved in carrageenan-induced inflammation.

#### 2.3.2. Inhibition of Lipoxygenase (LOX) Activity

Lipoxygenases (LOX) are a group of non-heme, iron-requiring enzymes involved in the synthesis of polyunsaturated fatty acid derivatives, such as lipoxins, leukotrienes, and hepoxilins [21].

Soybean LOX, because of structural and functional similarities with mammalian LOXs, is commonly used for the study of anti-inflammatory agents [22]. The ability of the compounds to inhibit soybean LOX, presented as IC_50_ values after 7 min incubation, is demonstrated in Table 4. The IC_50_ values of nordihydroguaiaretic acid (NDGA), a non-selective antioxidant that inhibits LOX by maintaining the active site iron in the reduced (Fe^+2^), inactive state [23], as well as of ibuprofen, ketoprofen, and naproxen as reference compounds are also shown in Table 4.

Compounds **16**, **20**, and **23** were not soluble in the reaction mixture at concentrations higher than those reported in Table 4. With an increase of substrate (linoleic acid) concentration above saturation, no inhibition was observed, indicating that the examined compounds are competitive inhibitors of LOX.

The new derivatives are only moderate or weak LOX inhibitors, as demonstrated in Table 4, although more active than the parent drugs. The most potent is compound **17** (IC_50_ 41 μM), which lacks any COX inhibitory activity and possesses only mild anti-inflammatory action.

#### 2.3.3. Effect on Lipid Peroxidation

Enzymatically inactivated rat hepatic microsomal membranes, rich in polyunsaturated fatty acids, were used and peroxidation was induced by Fe(II)-ascorbate. Compounds **21** and **22**, expected to exert antioxidant activity due to the tyrosine phenolic group, were tested. Their effect against lipid peroxidation, after 45 min of incubation, proved to be poor, possibly because of reduced accessibility of this group to the lipid substrate.

#### 2.3.4. Preliminary Stability Study

In a preliminary stability control experiment, compound **16**, with the highest anti-inflammatory activity, was dissolved in an acetone-water mixture and incubated for 72 h at 37 °C. Samples were tested by thin layer chromatography (TLC) at the time of the preparation and after 2.5, 5, 24, 48, and 72 h. The same experiment was repeated, but plasma from a healthy rat was added, and tested by TLC at the time of preparation and after 24 h of incubation at 37 °C. Only one spot, corresponding to the final compound **16**, was detected in all cases, indicating that the compound is not hydrolyzed under the described experimental conditions.

### 2.4. In Silico Study

In order to explain the difference of inhibition rate between the compounds, molecular docking studies were performed.

#### 2.4.1. Docking to COX-1 and COX-2

Results of molecular docking of compounds with crystal structures of COX-1 and COX-2 are given in Table 5.

**Figure 2:**
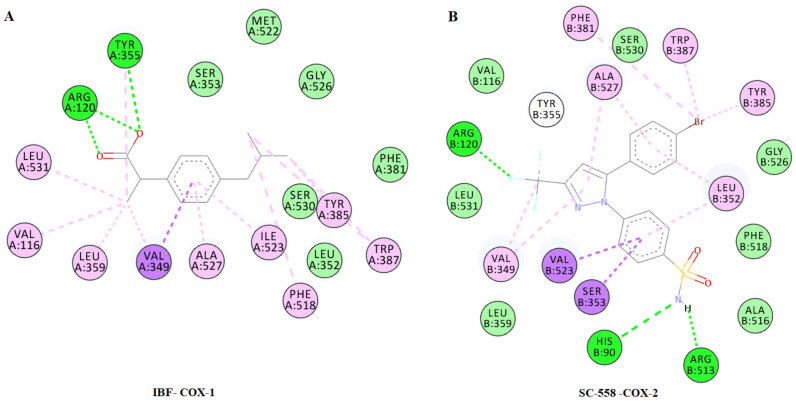
(**A**). Docking pose of co-crystallized ligand, ibuprofen (*S*) (IBF), in the COX-1 active site. (**B**) Docking pose of co-crystallized SC-558 inhibitor in the COX-2 active site.

**Figure 3:**
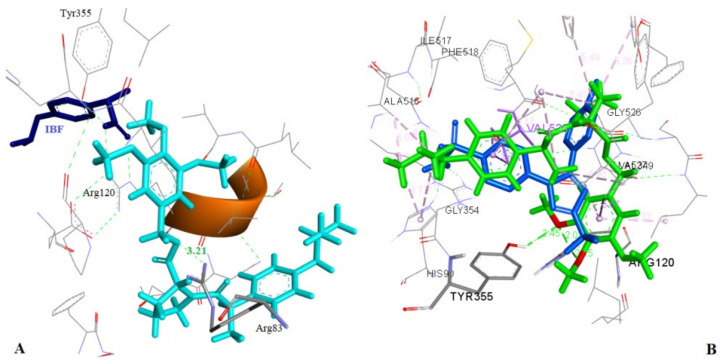
(**A**). Docked compound **19** (cyan) superimposed on the co-crystallized ibuprofen (blue) in the binding pocket of COX-1. (**B**) Docked compound **19** (green) superimposed on the co-crystallized SC-558 (blue) in the binding pocket of COX-2.

Molecular docking studies revealed that the co-crystallized ligand, ibuprofen (*S*), binds the COX-1 active site forming three hydrogen bonds through its carboxylate group, two with Arg120 and another with Tyr355 (Figure 2). Moreover, hydrophobic interactions with the residues Val349, Ile523, Ala527, Ser530, and Leu531 were detected. Compound **19** is placed inside the COX-1 enzyme adopting an unfavorable orientation for COX-1 inhibition, despite the hydrogen bond formed between residue Arg83 and oxygen of the methoxy group of compound **19** (Figure 3, Table 5).

SC-558 inhibitor binds to the active site of COX-2 by forming a weak H-bond between its trifluoromethyl group and the Arg120 side chain. Another two hydrogen bonds are formed through the interaction of the sulfonamide group with His90 and Arg513. This binding of the phenylsulphonamide group is responsible for the selectivity of SC-558 toward COX-2, since it binds to a pocket of COX-2, which is not accessible in COX-1 (Figure 2B). The phenyl ring of SC-558 also interacts hydrophobically with the residues Leu352, Ser353, Phe518, and Val523.

**Figure 4:**
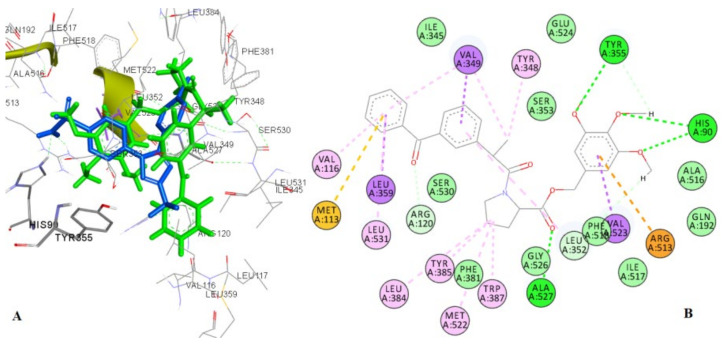
(**A**). Docked compound **19** (green) superimposed on the co-crystallized SC-558 (blue) in the binding pocket of COX-2. (**B**) 2D diagram of compound **19** in the binding pocket of COX-2. Hydrogen bonds are shown as green dotted lines.

Docking of the synthesized compounds to COX-2 displayed similar interactions with that of SC-558 inhibitor. In particularly, all compounds interacted with the residue Arg120 forming H-bond with their methoxy groups. The most active compound **19** interacts with His90 forming two hydrogen bonds with its methoxy substituents (Figure 4, Table 5). This interaction with His90 residue is probably responsible for the greater selectivity of **19** toward COX-2.

It is noteworthy to mention that all docked complexes showed greater selectivity toward COX-2 than COX-1, which is reflected by their lower binding score and of course their higher inhibitory action.

#### 2.4.2. Docking to the Active Site of 5-LOX

A theoretical study of the binding of the synthesized compounds to the active site of 5-LOX was performed. Compound **17** ((*S*)-3,4,5-trimethoxybenzyl 3-(2-(6-methoxynaphthalen-2-yl)propanamido)propanoate) with the highest inhibitory activity was studied in comparison with the known 5-LOX inhibitor, NDGA, used as reference compound.

Results of docking studies revealed that residue His367 is a common binding site for NDGA and compound **17**. This is very important because this residue, together with residue His372, plays a catalytic role in the active site of the 5-LOX enzyme, retaining iron [24].

**Figure 5:**
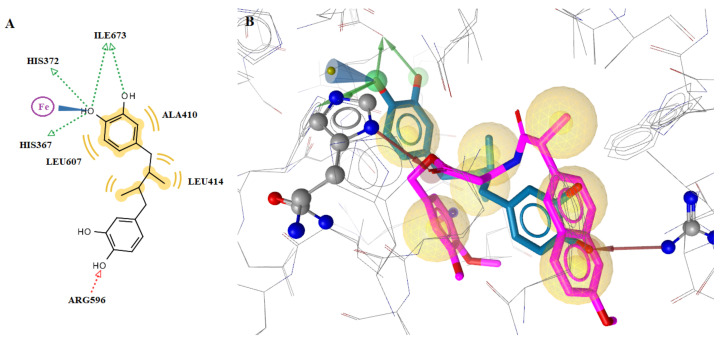
(**A**) 2D interaction diagram of the reference compound NDGA with the key amino acids in 5-LOX. (**B**) Superposition of compound **17** (magenta) bound to 5-LOX in comparison with NDGA (blue), with specific residues labeled. Active site zinc shown as yellow small sphere, red and green dotted arrows indicate H-bond, and yellow spheres hydrophobic interactions.

NDGA, used as a reference compound in the in vitro assay, as it is illustrated in Figure 5, forms five hydrogen bonds with the residual amino acids His372, His367, Arg596, and Ile673 of the active site of 5-LOX via its phenolic hydroxyl carriers. These residues play a significant role in the activity of the enzyme, because they either retain iron or form the α2-helix. Moreover, NDGA interacts hydrophobically with residues Leu607, Ala410, Leu414, and others (Figure 5). All these interactions stabilize the enzyme–inhibitor complex and explain its strong inhibitory activity (binding energy-12.96 kcal/mol, IC_50_ = 1.3 μM).

**Figure 6:**
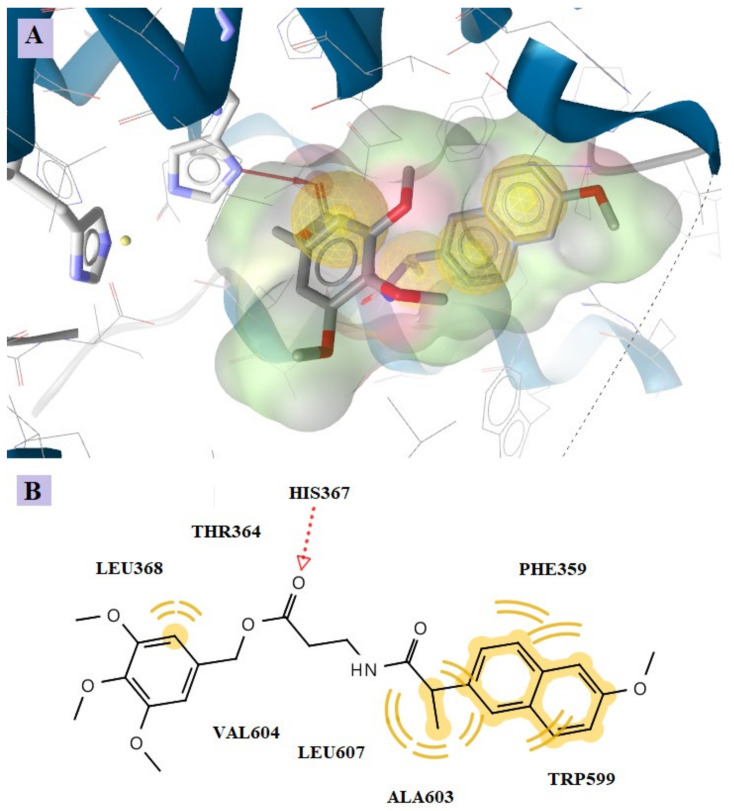
(**A**) Docking pose of most active compound **17** in 5-LOX enzyme. (**B**) 2D interaction diagram of compound **17** with the key amino acids in 5-LOX. Active site zinc shown as yellow small sphere, red dotted arrows indicate H-bond, and yellow spheres hydrophobic interactions.

The preeminence in inhibitory activity of 5-LOX of compound **17** (binding energy-8.76 kcal/mol, IC_50_ = 41 μM), among the tested compounds, can be elucidated by the fact that it forms a hydrogen bond between the oxygen of the carbonyl group and the residue His367, which participates in the retention of iron. Additionally, the presence of hydrophobic interactions with a number of residues such as Leu368, Thr364, Leu607, Trp599, and Phe359 probably increases the stability of the enzyme–inhibitor complex (Figure 6B).

For all the other compounds, binding energy ranged between −7.42 and −3.8 kcal/mol, and no hydrogen bonds were detected, especially with the crucial residues His367 or His372. Perhaps this is a reason for their reduced activity.

Concluding, the structure–activity relationship showed that, for a compound to be a good inhibitor of 5-LOX, the formation of a hydrogen bond with the residues His367 or His372 is essential, since they participate in the retention of iron at the active site of the enzyme.

## 3. Materials and Methods

### 3.1. General

Commercially available chemicals of the appropriate purity were purchased from Merck (Kenilworth, NJ, USA) or Sigma (St. Louis, MO, USA). The IR spectra were recorded on a Perkin Elmer Spectrum BX FT-IR spectrometer (Waltham, MA, USA). The ^1^H NMR and ^13^C NMR spectra were recorded using an AGILENT DD2-500 MHz (Santa Clara, CA, USA) spectrometer. Chemical shifts were reported in *δ* (ppm) and signals are as follows: s, singlet; d, doublet; t, triplet; m, multiplet. Melting points (m.p.) were determined with a MEL-TEMPII apparatus, Laboratory Devices, Sigma-Aldrich (Milwaukee, WI, USA) and were uncorrected. The microanalyses were performed on a Perkin-Elmer 2400 CHN elemental analyzer (Waltham, MA, USA). Thin-layer chromatography (TLC silica gel 60 F254 aluminum sheets, Merck (Kenilworth, NJ, USA) was used to follow the reactions and the spots were visualized under UV light. Ibuprofen and ketoprofen were racemates, naproxen was the *S*-enantiomer.

### 3.2. Synthesis

#### 3.2.1. Synthesis of Amino Acid Methyl Esters (as Hydrochlorides) **1**, **2**, **3** and **4**

(a) The corresponding amino acid (alpha-L-alanine, beta-alanine, 0.1 mol) was dissolved in 100 mL methanol and 0.2 mol of freshly distilled trimethylchlorosilane was added with stirring at room temperature. After the completion of the reaction (about 12 h), the solvent was evaporated under reduced pressure to give the esters **1** ((*S*)-1-methoxy-1-oxopropan-2-aminium chloride (*S*-alanine methyl ester hydrochloride)) and **2** (3-methoxy-3-oxopropan-1-aminium chloride (beta-alanine methyl ester hydrochloride)).
(b) 0.1 mol of amino acid (L-proline, L-tyrosine) was mixed with freshly distilled SOCl_2_ (0.3 mol) and Et_3_N (0.1 mol) in 100 mL methanol at room temperature. After the completion of the reaction (about 10 h), the mixture was neutralized with Et_3_N, the volatiles were removed under reduced pressure to give the amino acid methyl esters **3** ((*S*)-methyl pyrrolidine-2-carboxylate hydrochloride (L-proline methyl ester hydrochloride)) and **4** ((*S*)-methyl 2-amino-3-(4-hydroxyphenyl)propanoate hydrochloride (L-tyrosine methyl ester hydrochloride)).

Yields: 75–99% for both methods.

#### 3.2.2. General Method for the Synthesis of Amides **5**–**14**

The amino acid methyl ester hydrochloride (1 mol) was dissolved or suspended in dry chloroform and Et_3_N (1.1 mol) was added. Then, the corresponding acid (1.1 mol) and DCC (1.1 mol) were added slowly. The reaction mixture was stirred at room temperature overnight, filtered and washed with a 10% aqueous potassium carbonate solution. The organic layer was dried over sodium sulfate and concentrated. The residue was purified by flash chromatography, eluting with petroleum ether-ethyl acetate.

The obtained amides of the NSAID or indole-3-acetic acid with the amino acid methyl esters (10 mmol) were dissolved in a mixture of 100 mL dioxane and 100 mL aqueous 5% NaOH. The mixture was stirred for 1 h at ambient temperature. Dioxane was removed under reduced pressure and water (100 mL) was added. The mixture was acidified with 1N HCl and extracted with chloroform. The organic layer was dried (Na_2_SO_4_) and the final compounds (**5**–**14**) were purified by flash chromatography using mixtures of petroleum ether and ethyl acetate.

The following compounds are reported in the literature by others or by us: **5** (3-(2-(4-isobutylphenyl)propanamido)propanoic acid) [25]; **6** (as methyl ester), 3-(2-(3-benzoylphenyl)propanamido)propanoic acid [26]; **7** ((3-(2-(6-methoxynaphthalen-2-yl)propanamido)propanoic acid) [27]; **8** ((1-(2-(4-isobutylphenyl)propanoyl)pyrrolidine-2-carboxylic acid)) [28]; **9** (1-(2-(3-benzoylphenyl)propanoyl)pyrrolidine-2-carboxylic acid) [29]; **10** (1-(2-(6-methoxynaphthalen-2-yl)propanoyl)pyrrolidine-2-carboxylic acid) [11]; **11** (as methyl ester) (3-(4-hydroxyphenyl)-2-(2-(4-isobutylphenyl)propanamido)propanoic acid) [30]; **13** (2-(2-(1*H*-indol-3-yl)acetamido)propanoic acid) [31].

2-(2-(3-Benzoylphenyl)propanamido)-3-(4-hydroxyphenyl)propanoic acid (**12**) methyl ester.

Yield 78%. ^1^H-NMR: (CDCl_3_) *δ*: 1.61 (d, 3H, *J* = 7.2 Hz, -C**H_3_**), 2.98–3.11 (m, 2H, -C**H_2_**-CH-NH), 3.63–3.68 (m, 1H, C**H**CO-), 3.73 (s, 3H, COOC**H_3_**), 4.85–4.92 (m, 1H, -NHC**H**), 6.73 (d, 2H, *J* = 8.4 Hz, aromatic tyrosine **H_3_**, **H_5_**), 7.01 (d, 2H, *J* = 8.4 Hz, aromatic tyrosine **H_2_**, **H_5_**), 7.37–7.47 (m, 4H, benzaldehyde **H_3_**, **H_4_**, **H_5_**, isopropylphenyl **H_5_**), 7.62–7.80 (m, 5H, benzaldehyde **H_2_**, **H_6_**, isopropylphenyl **H_2_**, **H_4_**, **H_6_**).

1-(2-(1*H*-indol-3-yl)acetyl)pyrrolidine-2-carboxylic acid (**14**) methyl ester.

Yield 90% ^1^H-NMR: (CDCl_3_) *δ*: 2.00–2.22 (m, 3H, -NCH_2_C**H_2_**CH_2_CH- and -NCH_2_CH_2_C**H**_2_CH axial), 2.24–2.31 (m, 1H, -NCH_2_CH_2_C**H_2_**CH- equatorial), 3.53–3.59 (m, 2H, -NC**H_2_**CH_2_CH_2_CH-), 3.72 (s, 2H, C**H_2_**CONH-), 3.81 (s, 3H, COOC**H_3_**), 4.47–4.52 (m, 1H, -NCH_2_CH_2_CH**_2_**C**H**-), 7.06 (s, 1H, indole **H_2_**), 7.12–7.34 (m, 4H, indole **H_4_**, **H_5_**, **H_6_**, **H_7_**), 7.39 (s, 1H, indole-N**H**).

#### 3.2.3. General Method for the Synthesis of Compounds **15**–**24**

The corresponding acid **5**–**14** (1 mmol) and N,N′-dicyclohexylcarbodiimide (DCC, 1 mmol) were mixed in dry CHCl_3_ at ambient temperature. Then, 3,4,5-trimethoxybenzylalcohol (1.1 mmol) and N,N-dimethylaminopyridine (DMAP, 0.1 mmol) were added. The mixture was stirred for 4 h at ambient temperature, filtered and washed with a 10% aqueous potassium carbonate solution. The organic layer was dried over sodium sulfate, filtered and concentrated under reduced pressure. The residue was purified by flash chromatography, eluting with petroleum ether-ethyl acetate, to give the final compounds **15**–**24**.

*3,4,5-Trimethoxybenzyl 3-(2-(4-isobutylphenyl)propanamido)propanoate (15)*. It was isolated by flash chromatography (petroleum ether/ethyl acetate 5:1) as white solid, yield 69%, m.p. 73–75 °C. IR (nujol) ν: 1749, 1638 cm^−1^. ^1^H-NMR: (CDCl_3_) *δ*: 0.89 (d, 6H, *J* = 6.6 Hz, (C**H_3_**)_2_CHCH_2_-), 1.31, 1.35 (d, 3H, *J* = 7.2 Hz, -Ph-CH(C**H_3_**)CO-), 1.49, 1.50 (d, 2H, *J* = 7.2 Hz, (CH_3_)_2_CHC**H_2_**-), 1.81–1.88 (m, 1H, (CH_3_)_2_C**H**CH_2_-), 2.44 (d, 2H, *J* = 7.2 Hz, -NHCH_2_C**H_2_**-), 3.52–3.58, 4.55–4.62 (m, 2H, -NHC**H_2_**CH_2_-), 3.63 (q, 2H, *J* = 6.8 Hz, -Ph-C**H**(CH_3_)CO-), 3.84, 3.85, 3.86 (s, 9H, -Ph(OCH_3_)_3_), 5.05 (d, 2H, *J* = 3.4 Hz, -O-C**H_2_**-Ph(OCH_3_)_3_), 5.08 (s, 1H, CON**H**CH), 6.54 (d, 2H, *J* = 7.7 Hz, -CH_2_**Ph**(OCH_3_)_3_), 7.10 (d, 2H, *J* = 8.0 Hz, phenyl **H_3_**, **H_5_**), 7.18 (d, 2H, *J* = 8.0 Hz, phenyl **H_2_**, **H_6_**). ^13^C NMR (CDCl_3_) *δ*: 18.26 (1C, O=C-CH-**C**H_3_), 22.35 (2C, -CH**_2_**CH-(**C**H_3_)_2_), 30.14 (1C, -CH**_2_C**H-(CH_3_)_2_), 30.91 (1C, -NHCH_2_**C**H_2_-), 45.00 (1C, -NH**C**H_2_CH_2_-), 46.55 (1C, -**C**H_2_CH-(CH_3_)_2_), 48.20 (1C, -Ph-**C**H(CH_3_)CO-), 56.13 (2C, -O-**C**H_2_-Ph(**C_3_**_,_ **5**)OCH_3_), 60.81 (1C, -O-**C**H_2_-Ph(**C_4_**)OCH_3_), 67.19 (1C, -O-**C**H_2_-Ph(OCH_3_)_3_), 105.23 (2C, aromatic trimethoxy-benzyl **C_2_**, **C_6_**), 127.24, 127.38 (2C, aromatic phenyl **C_2_**, **C_6_**), 129.58 (2C, aromatic phenyl **C_3_**, **C_5_**), 130.90 (1C, aromatic trimethoxy-benzyl **C_1_**), 137.70 (1C, aromatic trimethoxy-benzyl **C_4_**), 138.15 (aromatic phenyl **C_1_**), 140.75 (aromatic phenyl **C_4_**), 153.32 (aromatic trimethoxy-benzyl **C_3_**, **C_5_**), 172.75 (1C, -NHCH_2_CH_2_**C**O-), 174.01 1C, -Ph-CH(CH_3_)**C**O-). Anal. calcd for C_26_H_35_NO_6_ (%): C 68.25; H 7.71; N 3.06. Found (%): C 68.34; H 7.64; N 2.83.

*3,4,5-Trimethoxybenzyl 3-(2-(3-benzoylphenyl)propanamido)propanoate (16)*. It was isolated by flash chromatography (petroleum ether/ethyl acetate 5:1) as colorless liquid, yield 59%, IR (nujol) ν: 1749, 1638 cm^−1^. ^1^H-NMR (CDCl_3_) *δ*: 1.52 (d, 3H, *J* = 7.1 Hz, -PhCH(C**H_3_**)CO-), 2.55 (dd, 2H, *J* = 11.2, 5.6 Hz, -NHCH_2_C**H_2_**-), 3.45–3.60 (m, 3H, -NHC**H_2_**CH_2_- and PhC**H**(CH_3_)CO-), 3.83, 3.85 (s, 9H, -PhO(C**H_3_**)**_3_**), 5.01 (d, 2H, *J* = 3.4 Hz, -OC**H_2_**Ph(OCH_3_)_3_), 6.01 (s, 1H, CON**H**CH), 6.55 (s, 2H, -OCH_2_ **Ph(**OCH_3_)_3_), 7.41–7.61 (m, 5H, benzyl **H_3_**, **H_4_**, **H_5_** and phenyl **H_5_**, **H_6_**), 7.66 (d, 1H, *J* = 7.6 Hz, phenyl **H_4_**), 7.72 (s, 1H, phenyl **H_2_**), 7.78–7.81 (m, 2H, benzoyl **H_2_**, **H_6_**). ^13^C NMR (CDCl_3_) *δ*: 18.55 (1C, O=C-CH-**C**H_3_), 33.98 (1C, -NHCH_2_**C**H_2_-), 35.06 (1C, -NH**C**H_2_CH_2_-), 46.96 (1C, -Ph-**C**H(CH_3_)CO-), 56.15 (2C, -O-**C**H_2_-Ph(**C_3_**_,_**C_5_**)OCH_3_), 60.62 (1C, -O-**C**H_2_-Ph(**C_4_**)OCH_3_), 66.82 (1C, -O-**C**H_2_-Ph(OCH_3_)_3_), 105.95 (2C, aromatic trimethoxy-benzyl **C_2_**, **C_6_**), 128.32 (1C, **C_2_** phenyl), 128.71 (1C, **C_4_** phenyl), 129.08, 129.02 (2C, **C_3_**, **C_5_** benzoyl), 130.04 (2C, **C_2_**, **C_6_** benzoyl), 130.99 (1C, **C_5_** phenyl), 131.44 (2C, **C_6_** phenyl, **C_4_** benzoyl), 132.95 (1C, trimethoxy-benzyl **C_1_**), 137.40 (1C, aromatic trimethoxy-benzyl **C_4_**), 138.06, 138.08 (2C, **C_3_** phenyl, **C_1_** benzoyl), 141.66 (1C, **C_1_** phenyl), 153.34 (aromatic trimethoxy-benzyl **C_3_**, **C_5_**), 172.26 (1C, -NHCH_2_CH_2_**C**O-), 173.90 (1C, -Ph-CH(CH_3_)**C**O-), 196.55 (1C, (Ph)_2_-**C**=O). Anal. calcd for C_29_H_31_NO_7_ (%): C 68.90; H 6.18; N 2.77. Found (%): C 68.50; H 5.99; N 2.81.

*3,4,5-Trimethoxybenzyl3-(2-(6-methoxynaphthalen-2-yl)propanamido) propanoate (17)*. It was isolated by flash chromatography (petroleum ether/ethyl acetate 5:1) as colorless liquid, yield 80% IR (nujol) ν: 1749, 1638 cm^−1^. ^1^H-NMR (CDCl_3_) *δ*: 1.56 (d, 3H, *J* = 7.1 Hz, -CHC**H_3_**CONH-), 2.53 (dd, 2H, *J* = 9.9, 6.0 Hz, -NHCH_2_C**H_2_**COO-), 3.47 (q, 2H, *J* = 6.1 Hz, -NHC**H_2_**CH_2_COO-), 3.63 (q, 1H, *J* = 7.1 Hz, -C**H**CH_3_CONH-), 3.80, 3.84 (s, 9H, -Ph(OC**H_3_**)_3_), 3.91 (s, 3H, -napthyl-OC**H_3_**), 4.90 (q, 2H, *J* = 12.1 Hz OC**H**_2_Ph(OCH_3_)_3_), 5.91 (s, 1H, CON**H**CH), 6.49 (s, 2H, -OCH_2_**Ph**(OCH**_3_**)_3_), 7.10 (d, 1H, *J* = 2.4 Hz, naphthyl C5), 7.14 (dd, 1H, *J* = 8.9, 2.5 Hz, naphthyl **C_7_**), 7.32 (dd, 1H, *J* = 8.5, 1.5 Hz, naphthyl **C_3_**), 7.62 (s, 1H, napthyl **C_1_**), 7.68 (d, 1H, *J* = 5.8 Hz, naphthyl **C_8_**), 7.69 (d, 1H, *J* = 6.2 Hz, naphthyl **C_4_**). ^13^C NMR (CDCl_3_) *δ*: 18.40 (1C, O=C-CH-**C**H_3_), 34.00 (1C, -NHCH_2_**C**H_2_-), 35.08 (1C, -NH**C**H_2_CH_2_-), 47.02 (1C, -Ph-**C**H(CH_3_)CO-), 55.30 (1C, -naphthyl-O**C**H_3_), 56.13 (2C, -O-CH_2_-Ph(**C_3_**_,_**C_5_**)OCH_3_), 60.82 (1C, -O-CH_2_-ph(**C_4_**)OCH_3_), 66.63 (1C, -O-**C**H_2_-Ph(OCH_3_)_3_), 105.41 (2C, aromatic trimethoxy-benzyl **C_2_**, **C_6_**), 105.60 (1C, naphthyl **C_5_**), 119.11 (1C, naphthyl **C_7_**), 126.00 (1C, naphthyl **C_1_**), 126.11 (1C, naphthyl **C8a**), 127.47 (1C, naphthyl **C_4_**), 128.94 (1C, naphthyl **C_3_**), 129.19 (1C, naphthyl **C_8_**), 131.08 (1C, aromatic trimethoxy-benzyl **C_1_**), 133.70 (1C, naphthyl **C4a**), 136.34 (1C, aromatic trimethoxy-benzyl **C_4_**), 138.21 (1C, naphthyl **C_2_**), 153.31 (aromatic trimethoxy-benzyl **C_3_**, **C_5_**), 157.70 (1C, naphthyl **C_6_**), 172.08 (1C, -NHCH_2_CH_2_**C**O-), 174.31 (1C, -Ph-CH(CH_3_)**C**O-). Anal. calcd for C_27_H_31_NO_7_ (%): C 67.34; H 6.49; N 2.91. Found (%): C 67.34; H 6.88; N 2.55.

*3,4,5-Trimethoxybenzyl 1-(2-(4-isobutylphenyl)propanoyl)pyrrolidine-2-carboxylate) (18)*. It was isolated as a diastereomer mixture by flash chromatography (petroleum ether/ethyl acetate 5:1). White solid, yield 55%, m.p. 85–87 °C. IR (nujol) ν: 1749, 1638 cm^−1^. ^1^H-NMR (CDCl_3_) *δ*: 0.89 (d, 6H, *J* = 6.6 Hz, (C**H_3_**)_2_CHCH_2_-), 1.42 (d, 3H, *J* = 6.8 Hz, -Ph-CH(C**H_3_**)CO-), 1.60–2.30 (m, 4H, (CH_3_)_2_C**H**CH_2_-, CH_2_C**H_2_**CH_2_CHCO-, CH_2_CH_2_C**H**_2_CHCO-axial, CH_2_CH_2_C**H**_2_CHCO-equatorial), 2.43 (d, 2H, *J* = 7.2 Hz, (CH_3_)_2_CHC**H_2_**-), 3.23–3.29 (m, 1H, -C**H_2_**CH_2_CH_2_CHCO- equatorial) 3.44–3.50, 3.63–3.80 (m, 3H, -C**H_2_**CH_2_CH_2_CHCO- axial, -PhC**H**(CH_3_)CO-), 3.84, 3.88 (s, 9H, -Ph(O**C**H_3_)_3_), 4.50 (dd, 1H, *J* = 8.5, 4.3 Hz, -CHCH_2_CH**_2_**C**H**CO-), 5.13 (s, 2H, -O-C**H_2_**-Ph-), 6.61 (s, 2H, -CH_2_**Ph**(OCH_3_)_3_), 7.07 (d, 2H, *J* = 8.1 Hz, phenyl **C_3_**, **C_5_**), 7.17 (d, 2H, *J* = 8.1 Hz, phenyl **C_2_**, **C_6_**). ^13^C NMR (CDCl_3_) *δ*: 20.05 (1C, O=C-CH-**C**H_3_), 22.38 (2C, -CH**_2_**CH-(**C**H_3_)_2_), 24.80, 25.69 (1C, CH_2_**C**H_2_CH_2_CHCO-), 29.03 (1C, CH_2_CH_2_**C**H_2_CHCO-), 30.15 (1C, -CH**_2_C**H-(CH_3_)_2_), 44.52 (1C, -Ph-**C**H(CH_3_)CO-), 45.01 (1C, -**C**H_2_CH-(CH_3_)_2_), 46.68 (1C, -NH**C**H_2_CH_2_-), 56.12 (2C, -O-CH_2_-Ph(**C_3_**_,**5**_)OCH_3_), 56.17 (1C, CH_2_CH_2_CH_2_**C**HCO-), 59.17 (1C, -O-CH_2_-Ph(**C_4_**)OCH_3_), 66.92 (1C, -O-**C**H_2_-Ph(OCH_3_)_3_), 105.18 (2C, aromatic trimethoxy-benzyl **C_2_**, **C_6_**), 127.19 (2C, aromatic phenyl **C_2_**, **C_6_**), 129.46 (2C, aromatic phenyl **C_3_**, **C_5_**), 131.41 (1C, aromatic trimethoxy-benzyl **C_1_**), 137.69 (1C, aromatic trimethoxy-benzyl **C_4_**), 138.32 (aromatic phenyl **C_1_**), 140.28 (aromatic phenyl **C_4_**), 153.28 (aromatic trimethoxy-benzyl **C_3_**, **C_5_**), 172.35 (1C, -NHCH_2_CH_2_**C**O-), 172.89 (1C, -Ph-CH(CH_3_)**C**O-). Anal. calcd for C_28_H_37_NO_6_ (%): C 69.54; H 7.71; N 2.90. Found (%): C 69.46; H 7.75; N 3.23.

*3,4,5-Trimethoxybenzyl 1-(2-(3-benzoylphenyl)propanoyl)pyrrolidine-2 carboxylate (19)*. It was isolated by flash chromatography (petroleum ether/ethyl acetate 5:1) as colorless liquid diastereomer mixture, yield 62%, IR (nujol) ν: 1749, 1638 cm^−1^. ^1^H-NMR (CDCl_3_) *δ*: 1.48 (d, 3H, *J* = 6.9 Hz, -PhCH(C**H_3_**)CO-), 1.81–2.10 (m, 4H, -CONCH_2_C**H_2_**C**H_2_**CH-CO-), 3.20–3.24, 3.70–3.75 (m, 2H, -CONC**H**_2_CH_2_CH_2_CHCO- axial and equatorial), 3.84, 3.88 (s, 9H, -PhO(C**H_3_**)**_3_**), 3.90 (q, 1H, *J* = 6.9 Hz, PhC**H**(CH_3_)CO-), 4.50 (dd, 1H, *J* = 8.4, 4.2 Hz, -CONCH_2_CH_2_CH_2_C**H**-CO-), 5.13 (s, 2H, -OC**H_2_**PhO(CH_3_)_3_), 6.60 (s, 2H, -OCH_2_**Ph**O(CH_3_)_3_), 7.32–7.54 (m, 3H, **H_3_**, **H_4_**, **H_5_** benzoyl), 7.56–7.74 (m, 3H, **H_1_**, **H_5_**, **H_6_** phenyl), 7.72–7.86 (m, 3H, **H_2_**, **H_6_** benzoyl, **H_4_** phenyl). ^13^C NMR (CDCl_3_) *δ*: 19.93 (1C, O=C-CH-**C**H_3_), 24.79 (1C, CH_2_**C**H_2_CH_2_CHCO-), 29.02 (1C, CH_2_CH_2_**C**H_2_CHCO-), 44.59 (1C, -Ph-**C**H(CH_3_)CO-), 46.96 (1C, **C**H_2_CH_2_CH_2_CHCO-), 56.16 (2C, -O-CH_2_-ph(**C_3,5_**)OCH_3_), 59.20 (1C, CH_2_CH_2_CH_2_**C**HCO-), 60.81 (1C, -O-CH_2_-Ph(**C_4_**)OCH_3_), 66.99 (1C, -O-**C**H_2_-Ph(OCH_3_)_3_), 105.17 (2C, aromatic trimethoxy-benzyl **C_2_**, **C_6_**), 128.30 (1C, **C_2_** phenyl), 128.76 (1C, **C_4_** phenyl), 128.91, 129.15 (2C, **C_3_**, **C_5_** benzoyl), 130.04 (2C, **C_2_**, **C_6_** benzoyl), 131.36 (1C, **C_5_** phenyl), 131.44 (2C, **C_6_** phenyl, **C_4_** benzoyl), 132.57 (1C, trimethoxy-benzyl **C_1_**), 137.42 (1C, aromatic trimethoxy-benzyl **C_4_**), 137.84, 138.01 (2C, **C_3_** phenyl, **C_1_** benzoyl), 141.53 (1C, **C_1_** phenyl), 153.29 (aromatic trimethoxy-benzyl **C_3_**, **C_5_**), 172.12 (1C, CH_2_CH_2_CH_2_CH**C**O-), 172.31 (1C, -Ph-CH(CH_3_)**C**O-), 196.54 (1C, (Ph)_2_-**C**=O). Anal. calcd for C_31_H_33_NO_7_ (%): C 70.04; H 6.26; N 2.63. Found (%): C 70.08; H 6.27; N 3.05.

*3,4,5-Trimethoxybenzyl1-(2-(6-methoxynaphthalen-2-yl)propanoyl) pyrrolidine-2-carboxylate (20)*. It was isolated by flash chromatography (petroleum ether/ethyl acetate 5:1) as white solid, yield 78%, m.p. 50–53 °C. IR (nujol) ν: 1749, 1638 cm^−1^. ^1^H-NMR (CDCl_3_) *δ*: 1.48 (d, 3H, *J* = 6.9 Hz, -CHC**H_3_**CONH-), 1.78–1.91 (m, 4H, -NCH_2_C**H_2_**C**H_2_**CH), 2.04 (s, 1H, -C-O**H**) 3.19–3.24 (m, 2H, -NC**H**_2_CH_2_CH_2_CH) 3.45–3.57 (m, 2H, -NC**H**_2_CH_2_CH_2_CH-), 3.80 (s, 6H, -Ph(**C_3_**,**C_5_**)OC**H_3_**), 3.82 (s, 3H, -Ph(**C_4_**)OC**H_3_**), 3.90 (s, 3H, C**H_3_**O-naphthyl-), 4.12 (q, 1H, *J* = 7.1 Hz, -C**H**CH_3_CONH-) 4.61 (dd, 1H, *J* = 8.5, 4.3 Hz, -NCH_2_CH_2_CH_2_C**H**-), 5.08, 5.14 (d, 2H, *J* = 12.3 Hz, -OC**H**_2_Ph(OCH_3_)_3_), 6.54 (s, 2H, -OCH_2_**Ph**(OCH**_3_**)_3_), 6.64 (s, 1H, -N-), 7.09 (d, 1H, *J* = 3.3 Hz, naphthyl **H_5_**), 7.11 (d, 1H, *J* = 2.5 Hz, naphthyl **H_7_**), 7.34 (d, 1H, *J* = 8.4 Hz, naphthyl **H_3_**), 7.59 (s, 1H, naphthyl **H_1_**), 7.62 (d, 1H, *J* = 3.2 Hz, naphthyl **H_8_**), 7.64 (d, 1H, *J* = 8.7 Hz, naphthyl **H_4_**). ^13^C NMR (CDCl_3_) *δ*: 20.24 (1C, O=C-CH-**C**H_3_), 24.91 (1C, -NCH_2_**C**H_2_CH_2_CH-), 29.05 (1C, -NCH_2_CH_2_**C**H_2_CH), 44.86 (1C, -Ph-**C**H(CH_3_)CO-), 46.83 (1C, -N**C**H_2_CH_2_CH_2_CH-), 55.29 (1C, -napthyl-O**C**H_3_), 56.06 (2C, -O-CH_2_-Ph(**C_3_**_,**5**_)OCH_3_), 59.24 (1C, -N**C**H_2_CH_2_CH_2_CH-), 60.79 (1C, -O-CH_2_-Ph(**C_4_**)OCH_3_), 66.70 (1C, -O-**C**H_2_-Ph(OCH_3_)_3_), 104.99 (2C, aromatic trimethoxy-benzyl **C_2_**, **C_6_**), 105.57, 105.68 (1C, naphthyl **C_5_**), 118.90 (1C, naphthyl **C_7_**), 126.01 (1C, naphthyl **C_1_**), 126.38 (1C, naphthyl **C8a**), 127.27 (1C, napthyl **C_4_**), 129.02 (1C, naphthyl **C_3_**), 129.19 (1C, naphthyl **C_8_**), 131.52 (1C, aromatic trimethoxy-benzyl **C_1_**), 133.39 (1C, naphthyl **C4a**), 136.27 (1C, aromatic trimethoxy-benzyl **C_4_**), 137.71 (1C, naphthyl **C_2_**), 153.25 (aromatic trimethoxy-benzyl **C_3_**, **C_5_**), 157.51 (1C, naphthyl **C_6_**), 172.03 (1C, -NHCH_2_CH_2_**C**O-), 172.46 1C, -Ph-CH(CH_3_)**C**O-). Anal. calcd for C_29_H_33_NO_7_ (%): C 68.62; H 6.55; N 2.76. Found (%): C 68.39; H 6.57; N 3.16.

*3,4,5-Trimethoxybenzyl3-(4-hydroxyphenyl)-2-(2(4-isobutylphenyl)propanamido) propanoate (21)*. It was isolated as a diastereomer mixture by flash chromatography (petroleum ether/ethyl acetate 5:1). White solid, yield 45%, m.p. 86–88 °C. IR (nujol) ν: 1749, 1638 cm^−1^. ^1^H-NMR (CDCl_3_) *δ*: 1.07 (d, 6H, *J* = 6.6 Hz, (C**H_3_**)**_2_**CHCH_2_Ph-), 1.90 (d, 3H, *J* = 6.8 Hz, -Ph-CH(C**H_3_**)CO-), 2.07–2.13 (m, 1H, (CH_3_)_2_C**H**CH_2_-), 2.56 (d, 2H, *J* = 7.2 Hz, (CH_3_)_2_CHC**H_2_**-), 3.88–3.93, 3.18–3.22 (m, 3H, -CHC**H_2_**(PhOH)-), 3.79–3.84 (m, 1H, -PhC**H**(CH_3_)CO-), 3.78, 3.82 (s, 9H, -Ph(OCH_3_)_3_), 4.86 (s, 1H, -CON**H**-), 5.02–5.07 (m, 1H, -C**H**CH_2_(PhOH)), 5.35 (s, 2H, (-O-C**H_2_**-ph-)), 6.73 (s, 2H, -CH_2_**Ph**(OCH_3_)_3_), 6.73 (d, 2H, *J* = 8.4 Hz **H_3_**, **H_5_** phenol), 6.95 (d, 2H, *J* = 8.4 Hz, **H_2_**, **H_6_** phenol), 7.21 (d, 2H, *J* = 8.0 Hz, phenyl **H_3_**, **H_5_**), 7.39 (d, 2H, *J* = 8.0 Hz, phenyl **H_2_**, **H_6_**). ^13^C NMR (CDCl_3_) *δ*: 16.78 (1C, O=C-CH-**C**H_3_), 22.18 (2C, -CH**_2_**CH-(**C**H_3_)_2_), 27.62 (1C, -CH**_2_C**H-(CH_3_)_2_), 37.62 (2C, -CH**C**H_2_(PhOH)), 45.74 (1C, -Ph-**C**H(CH_3_)CO-), 46.49 (1C, -**C**H_2_CH-(CH_3_)_2_), 54.84 (1C, -**C**HCH_2_(PhOH)), 56.78 (2C, -O-CH_2_-Ph(**C_3_**_,**5**_)O**C**H_3_), 60.65 (1C, -O-CH_2_-Ph(**C**_4_)O**C**H_3_), 67.12 (1C, -O-**C**H_2_-Ph(OCH_3_)_3_), 106.61 (2C, aromatic trimethoxy-benzyl **C_2_**, **C_6_**), 115.90 (2C, -CHCH_2_Ph(**C_3_**,**C_5_**)OH), 124.76 (2C, aromatic phenyl **C_2_**, **C_6_**), 129.74 (1C, -CHCH_2_Ph(**C_1_)**OH), 130.62 (2C, CHCH_2_Ph(**C_2_**,**C_6_**)OH), 131.54 (2C, aromatic phenyl **C_3_**, **C_5_**), 135.13 (1C, aromatic trimethoxy-benzyl **C_1_**), 139.22 (1C, aromatic trimethoxy-benzyl **C_4_**), 141.11 (aromatic phenyl **C_1_**), 142.56 (aromatic phenyl **C_4_**), 154.90 (aromatic trimethoxy-benzyl **C_3_**, **C_5_**), 155.93 (aromatic phenyl**C_4_** (OH)), 171.61 (1C, -NHCH_2_CH_2_**C**O-), 176.02 (1C, -Ph-CH(CH_3_)**C**O-). Anal. calcd for C_32_H_39_NO_7_ (%): C 69.92; H 7.50; N 2.55. Found (%): C 69.53; H 7.44; N 2.67.

*3,4,5-Trimethoxybenzyl 2-(2-(3-benzoylphenyl)propanamido)-3-(4-hydroxyphenyl) propanoate (22)*. It was isolated as a diastereomer mixture by flash chromatography (petroleum ether/ethyl acetate 5:1). White solid, yield 43%, m.p. 68–70 °C. IR (nujol) ν: 1749, 1638 cm^−1^. ^1^H-NMR (CDCl_3_) *δ*: 1.49, 1.81 (d, 3H, *J* = 7.0 Hz, -PhCH(C**H_3_**)CO-), 2.88–3.03 (m, 2H, CHC**H_2_**PhOH), 3.58, 3.63 (q, 1H, *J* = 7.1 Hz PhC**H**(CH_3_)CO-), 3.82, 3.84 (s, 9H, -PhO(C**H_3_**)**_3_**), 4.76–4.86 (m, 1H, NHC**H**CH_2_Ph(OH)), 4.97–5.13 (m, 2H, -OC**H_2_**PhO(CH_3_)_3_), 5.83, 5.85 (d, 1H *J* = 5.2 Hz, CON**H**-), 6.23 (s, 1H, PhO**H**), 6.48, 6.58 (d, 2H, *J* = 8.4 Hz **H_3_**, **H5** phenol), 6.55, 6.51 (s, 2H, -OCH_2_**Ph**O(CH_3_)_3_), 6.67 (d, 2H, *J* = 8.4 Hz, **H_2_**, **H_6_** phenol), 7.37–7.50 (m, 5H, **H_3_**, **H_4_**, **H_5_** benzyl, **H_5_**, **H_6_** phenyl), 7.57–7.79 (m, 4H, **H_4_**, **H_2_** phenyl, **H_2_**, **H_6_** benzoyl). ^13^C NMR (CDCl_3_) *δ*: 18.17, 18.25 (1C, O=C-CH-**C**H_3_), 36.71 (1C, -Ph-**C**H(CH_3_)CO), 46.80 (1C, **C**H_2_CH_2_CH_2_CHCO-), 53.00, 53.20 (2C, -O-**C**H_2_-Ph(**C_3,5_**)OCH_3_), 56.15 (1C, -NH**C**HCO-), 60.85 (1C, -O-**C**H_2_-Ph(C4)OCH_3_), 67.48, 67.53 (1C, -O-**C**H_2_-Ph(OCH_3_)_3_), 105.92 (2C, aromatic trimethoxy-benzyl **C_2_**, **C_6_**), 115.53 (2C, **C_3_**, **C_5_** phenol), 126.79 (1C, **C_2_** phenyl), 128.41 (1C, **C_4_** phenyl), 128.92 (1C, **C_1_** phenol), 129.24 (2C, **C_3_**, **C_5_** benzoyl), 130.11 (2C, **C_2_**, **C_6_** benzoyl), 130.20 (2C, **C_2_**, **C_6_** phenol), 131.55, 131.63 (1C, **C_5_** phenyl), 132.68, 132.78 (2C, **C_6_** phenyl, **C_4_** benzoyl), 137.21, 137.25 (1C, trimethoxy-benzyl **C_1_**), 138.06, 138.01 (2C, **C_3_** phenyl, **C_1_** benzoyl), 140.77 (1C, aromatic trimethoxy-benzyl **C_4_**), 141.29 (1C, phenyl **C_1_**), 153.28 (aromatic trimethoxy-benzyl **C_3_**, **C_5_**), 155.13, 155.20 (1C, phenol **C_4_**), 171.27, 171.43 (1C, -NHCH**C**O-), 172.95, 173.30 (1C, -Ph-CH(CH_3_)**C**O-), 196.74, 196.99 (1C, (Ph)_2_-**C**=O). Anal. calcd for C_35_H_35_NO_8_ (%): C 70.34; H 5.90; N 2.34. Found (%): C 70.11; H 6.13; N 2.30.

*3,4,5-Trimethoxybenzyl 2-(2-(1H-indol-3-yl)acetamido)propanoate (23)*. It was isolated, by flash chromatography (petroleum ether/ethyl acetate 1:1) as yellow liquid, yield 75%, IR (nujol) ν: 1749, 1638 cm^−1^. ^1^H-NMR (CDCl_3_) *δ*: 2.52 (d, 3H, *J* = 7.1 Hz, -NHCH(C**H_3_**)CO-), 3.46 (q, 1H, *J* = 7.1 Hz, -NHC**H**(CH_3_)CO-), 3.69 (s, 2H, -C**H_2_**CONH-), 3.85 (s, 9H, -Ph(OC**H_3_**)**_3_**), 4.88 (s, 2H, -OC**H_2_**Ph(OCH_3_)_3_), 6.16 (s, 1H, CON**H**CH), 6.52 (s, 2H, -OCH_2_**Ph**(OCH_3_)_3_), 7.02 (s, 1H, indole **H_2_**), 7.12 (t, 1H, *J* = 7.1 Hz indole **H_5_**), 7.21 (t, 1H, *J*=7.1 Hz, indole **H_6_**), 7.39 (d, 1H, *J* = 7.8 Hz, indole **H_4_**), 7.51 (d, 1H, *J* = 7.8 Hz, indole **H_7_**), 8.35 (s, 1H, indole N**H**). ^13^C NMR (CDCl_3_) *δ*: 33.37 (1C, -NHCH(**C**H_3_)CO-), 34.12 (1C, -**C**H_2_CONH-), 35.00 (1C, NH**C**H(CH_3_)CO-), 56.19 (2C, -O-CH_2_-Ph(**C_3_**_,**5**_)OCH_3_), 60.86 (1C, -O-CH_2_-Ph(**C_4_**)O**C**H_3_), 66.65 (1C, -O-**C**H_2_-Ph(OCH_3_)_3_), 105.65 (2C, aromatic trimethoxy-benzyl **C_2_**, **C_6_**], 108.63 (1C, indole **C_3_**], 111.45 (1C, indole **C_7_**], 118.54 (1C, indole **C_4_**], 119.90 (1C, indole **C_5_**], 122.45 (1C, indole **C_2_**], 123.76 (1C, indole **C_6_**], 126.94 (1C, aromatic trimethoxy-benzyl **C_1_**], 131.21 (2C, indole **C3a**, **C7a**], 136.40 (1C, aromatic trimethoxy-benzyl **C_4_**], 153.27 (aromatic trimethoxy-benzyl **C_3_**, **C_5_**], 171.72 (1C, -NHCH_2_**C**O-], 171.90 (1C, Ph-**C**H_2_**C**O-]. Anal. calcd for C_23_H_26_N_2_O_6_ (%): C 64.78; H 6.15; N 6.57. Found (%): C 64.37; H 6.33; N 7.03.

*3,4,5-Trimethoxybenzyl1-(2-(1H-indol-3-yl)acetyl)pyrrolidine-2carboxylate (24)*. It was isolated as white solid by flash chromatography (petroleum ether/ethyl acetate 1:1), yield 75%, m.p. 58–60 °C. IR (nujol) ν: 1749, 1638 cm^−1^. ^1^H-NMR (CDCl_3_) *δ*: 1.85–2.26 (m, 4H, -NCH_2_C**H_2_**C**H_2_**CH-), 3.55–3.73 (m, 2H, -NC**H_2_**CH_2_CH_2_CH-), 3.79 (s, 2H, C**H_2_**CONH-), 3.81, 3.83 (s, 9H, -Ph(OC**H_3_**)**_3_**), 4.60 (dd, 1H, *J* = 8.6, 3.6 Hz, -NCH_2_CH_2_CH**_2_**C**H**-), 5.13 (s, 2H, -OC**H_2_**Ph(OCH_3_)_3_), 6.55 (s, 2H, -OCH_2_**Ph**(OCH_3_)_3_), 7.01–7.19 (m, 3H, indole **C_2_**, **C_5_**, **C_6_**), 7.34 (d, 1H, J = 8.1 Hz, indole **H_4_**), 7.58 (d, 1H, J = 7.9 Hz, indole **H_7_**), 8.34 (s, 1H, indole N**H**). ^13^C NMR (CDCl_3_) *δ*: 24.94 (1C, -NCH_2_**C**H_2_CH_2_CH-), 29.29 (1C, -NCH_2_CH_2_**C**H_2_CH), 32.09 (1C, -**C**H_2_CONH-), 47.39 (1C, -N**C**H_2_CH_2_CH_2_CH-), 56.14 (2C, -O-CH_2_-Ph(**C_3_**_,**5**_)OCH_3_), 59.01 (1C, -NCH_2_CH_2_CH_2_**C**H-), 60.82 (1C, -O-CH_2_-Ph(**C**_4_)O**C**H_3_), 66.97 (1C, -O-**C**H_2_-Ph(OCH_3_)_3_), 105.18 (2C, aromatic trimethoxy-benzyl **C_2_**, **C_6_**), 108.41 (1C, indole **C_3_**), 111.22 (1C, indole **C_7_**), 118.51 (1C, indole **C_4_**), 119.43 (1C, indole **C_5_**), 122.02 (1C, indole **C_2_**), 122.85 (1C, indole **C_6_**), 131.37 (1C, aromatic trimethoxy-benzyl **C_1_**), 136.13 (2C, indole), 137.78 (1C, aromatic trimethoxy-benzyl **C_4_**), 153.27 (aromatic trimethoxy-benzyl **C_3_**, **C_5_**), 170.44 (1C, -NHCH_2_**C**O-), 172.41 (1C, Ph-**C**H_2_**C**O-). Anal. calcd for C_25_H_28_N_2_O_6_ (%): C 66.36; H 6.24; N 6.19. Found (%):C 66.87; H 6.15; N 6.69.

### 3.3. Biological Evaluation

κ-Carrageenan and lipoxygenase type I-B from soybean were purchased from Sigma (St. Louis, MO, USA). COX inhibition was estimated using the “COX Inhibitor Screening Assay” kit (Cayman Chemical Co., Ann Arbor, MI, USA). For the in vivo experiments, female Wistar rats (160–180 g, 4–5 months old) were kept in the Centre of the School of Veterinary Medicine (EL54 BIO42), Aristotelian University of Thessaloniki, which is registered by the official state veterinary authorities (presidential degree 56/2013, in harmonization with the European Directive 2010/63/EEC). The Animal Ethics Committee of the Prefecture of Central Macedonia (no. 270079/2500) approved the experimental protocols.

#### 3.3.1. Effect on Carrageenan-Induced Rat Paw Edema

Total of 0.1 mL of an aqueous solution of carrageenan (1% *w*/*v*) was injected i.d. into the right hind paw of rats, with the left paw serving as control. The tested compounds (suspended in water with a few drops of Tween 80) were given i.p. (0.15 mmol/kg) 5 min before the carrageenan administration. After 3.5 h, the hind paws were excised and weighed separately. The produced edema was estimated as paw weight increase [17].

#### 3.3.2. Inhibition of COX-1 and COX-2 Activity

The effect of compounds on COX-1 and COX-2 activity was measured using a commercial kit and applying the instructions of the manufacturer. The kit uses ovine COX-1 and human recombinant COX-2 enzymes. The assay measures PGF_2a_ produced by SnCl_2_ reduction of COX-derived PGH_2_. The prostanoid product was quantified via enzyme immunoassay using a broadly specific antibody that binds to all the major prostaglandin compounds.

#### 3.3.3. Inhibition of LOX Activity

The reaction mixture contained (final concentration) the test compounds, dissolved in ethanol (10–300 μM), or the solvent (control), soybean LOX, dissolved in 0.9% NaCl solution (250 u/mL) and sodium linoleate (100 μM), in Tris–HCl buffer, pH 9.0. The reaction was followed for 7 min at 28 °C, recording the absorbance (234 nm) of a conjugated diene structure, due to the formation of 13-hydroperoxy-linoleic acid. The performance of the assay was verified using NDGA as a reference. For the estimation of the type of inhibition, the above experiments were repeated, using 1 mM sodium linoleate, which is higher than the saturating substrate concentration [32].

#### 3.3.4. Effect on Lipid Peroxidation

The peroxidation of heat-inactivated (90°, 90 s) rat liver microsomal fraction was induced by ascorbic acid (0.2 mM) and ferrous sulphate (10 μΜ). The studied compounds, in dimethylsulfoxide, were added at 1 mM. Aliquots were taken from the incubation mixture (37 °C) for 45 min. Lipid peroxidation was assessed spectrophotometrically (535/600 nm) as 2-thiobarbituric acid reactive material [32].

#### 3.3.5. Preliminary Stability Study

A solution of **16** in a 4:1 acetone–water mixture (1 mM) was prepared and 5 μL of this were applied on a TLC silica gel aluminum sheet and developed with petroleum ether-ethyl acetate (1:1), at the time of preparation and after 2.5, 5, 24, 48, and 72 h of incubation (37 °C). The same experiment was repeated, adding 1 mL of rat plasma. TLC was performed at the time of preparation and after 24 h.

### 3.4. In Silico Study

Molecular modeling studies were performed using the AutoDock 4.2 software [33]. X-ray crystal structures of COX-1 (PDB code: 1EQG) [34], COX-2 (PDB code: 1CX2) [6] with bound inhibitors and the human 5-LOX (PDB ID: 6N2W) [23] bound to NDGA, were retrieved from Brookhaven Protein Data Bank (PDB). The resulting poses and potential interactions were visualized using the Discovery studio visualizer version 4.0 (BIOVIA, San Diego, CA, USA). Moreover, in order to validate the accuracy of the docking program AutoDock 4.2, the co-crystallized ligands, ibuprofen, SC-558, and NDGA were docked into the active site of COX-1, COX-2, and 5-LOX enzymes respectively. The results revealed that the docked ligands ibuprofen (*S*), SC-558, and NDGA were exactly superimposed on the co-crystallized bound ones with a root mean square deviation value (RMSD) of 0.93 Å and 0.35 Å and 0.67 Å respectively. All the procedures were carried out as described in our previous work [35].

## 4. Conclusions

The molecular modifications performed on the structures of some classic NSAIDs resulted in the enhancement of the anti-inflammatory activity, compared with the parent compounds, and, in most cases, in a higher inhibitory activity toward COX-2 than COX-1. Molecular docking studies, used as a predictive tool, seem to agree with the experimental results. The carboxylic group of NSAID, not necessary for anti-inflammatory activity, represents a convenient means for derivatization toward more active and selective anti-inflammatory agents.

## Figures and Tables

**Table 1:** Effect of the synthesized compounds and their parent acids on carrageenan-induced rat paw edema. The effect on edema is expressed as percent inhibition of edema in comparison to controls, which received carrageenan only. All compounds were administered i.p. (0.15 mmol/kg). Each value represents the mean obtained from five female rats (160–180 g b.w.), and standard deviation is always within 10% of the mean value.

Compound	% Edema Inhibition	Compound	% Edema Inhibition
Ibuprofen	36 **	**22**	59 ***
**15**	35 *	Naproxen	11 *
**18**	49 ***	**17**	23 *
**21**	66.5 ***	**20**	37 *
Ketoprofen	47 **	Indole-3-acetic acid	0
**16**	91 ***	**23**	40.5 *
**19**	51 **	**24**	0

*** *p* < 0.0001; ** *p* < 0.0005; * *p* < 0.005, Student’s *t*-test.

**Table 2:** Effect of methyl esters on carrageenan-induced rat paw edema. The effect on edema is expressed as percent inhibition of edema in comparison to controls, which received carrageenan only. All compounds were administered i.p. (0.15 mmol/kg). Each value represents the mean obtained from five female rats (160–180 g b.w.), and standard deviation is always within 10% of the mean value.

No	Compound	% Edema Inhibition	No	Compound	% Edema Inhibition
**5a**	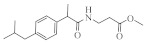	14 *	**13a**	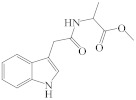	18 *
**8a**	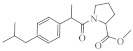	20 *	**14a**	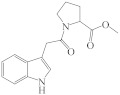	0

* *p* < 0.005, Student’s *t*-test.

**Table 3:** Effect of compounds on cyclooxygenase (COX)-1 and -2 activity and their molecular volume (MV).

Compound	COX-1 % Inhibition	COX-2 % Inhibition	ΜV * (Å^3^)
Ibuprofen	68	46	211.2
**18**	-	57	465.2
**21**	51	67	521.4
Ketoprofen	94	49	234.8
**16**	28	56	465.6
**19**	57	94	488.8
**22**	-	-	545.1
Naproxen	34	17	214.0
**17**	-	-	444.8
**20**	-	14	467.9

Each determination was performed in triplicate and SD values are between 2 and 4% of the mean value. * Calculated using Molinspiration Cheminformatics 2021.

**Table 4:** Effect of synthesized compounds, ibuprofen, ketoprofen, naproxen, and NDGA on lipoxygenase.

Compound	IC_50_ (μM) *	Compound	IC_50_ (μM) *
Ibuprofen	200	**22**	85
**15**	160	Naproxen	218
**18**	83	**17**	41
**21**	64	**20**	b
Ketoprofen	220	**23**	c
**16**	a	**24**	128
**19**	140	NDGA	1.3

*: After 7 min of incubation. NDGA: nordihydroguaiaretic acid. Each determination was performed in triplicate and SD values are between 2 and 4% of the mean value. a: 20% inhibition at 150 μΜ; b: 40% inhibition at 100 μΜ; c: 15% inhibition at 150 μΜ.

**Table 5:** Docking-assisted predicted values.

Compound	Ovine COX-1 (PDB:1EQG)		Mus musculus COX-2 (PDB: 1CX2)	
	Binding Free Energy (kcal/mol)	Hydrogen Bonds	Binding Free Energy (kcal/mol)	Hydrogen Bonds
**15**	−6.36	O-Arg83	−8.70	O-Arg120, O-Arg120, O-Tyr355
**16**	−5.90	O-Arg120	−8.54	O-Arg120, O-Tyr355
**18**	−5.52	-	−8.41	O-Arg120, O-Tyr355
**19**	−7.60	O-Arg120, O-Tyr355	−10.25	O-His90, O-His90, O-Tyr355, O-Ala527
**20**	−5.14	-	−6.72	O-Arg120
**21**	−7.48	O-Arg120, O-Arg120	−8.02	O-Arg120, O-Tyr355
**Ketoprofen**	−9.53	O-Arg120, O-Arg120, H-Tyr355	−8.11	O-Arg120, H-Tyr355
**Ibuprofen**	−9.22	O-Arg120, O-Arg120, O-Tyr355	-	-
**SC-558**	-	-	−9.17	O-His90, F-Arg120, H-Arg513

SC-558: 4-[5-(4-bromophenyl)-3-(trifluoromethyl)-1*H*-pyrazol-1-yl] benzenesulfonamide, selective COX-2 inhibitor used as reference compound.

## Data Availability

The data presented in this study are available on request from the corresponding author.

## Sample Availability

Not available.
